# Sex Differences in Spontaneous Degranulation Activity of Intrahepatic Natural Killer Cells during Chronic Hepatitis B: Association with Estradiol Levels

**DOI:** 10.1155/2017/3214917

**Published:** 2017-04-02

**Authors:** Zuzana Macek Jilkova, Thomas Decaens, Alice Marlu, Hélène Marche, Evelyne Jouvin-Marche, Patrice N. Marche

**Affiliations:** ^1^Université Grenoble-Alpes, IAB, 38000 Grenoble, France; ^2^INSERM U1209, 38000 Grenoble, France; ^3^Département d'Hépato-Gastro-Entérologie, CHU-Grenoble Alpes, 30700 La Tronche, France

## Abstract

Major sex differences are observed in the prevalence, intensity, and severity of hepatitis B virus (HBV) infection. Here, we investigated degranulation activity of circulating and intrahepatic natural killer (NK) cells from HBV and HCV chronically infected patients before any treatment (*n* = 125). The frequency of CD107^+^ NK cells in the female liver was significantly higher compared to that in males during chronic HBV infection (*p* = 0.002) and correlated with the plasma levels of estradiol (correlation coefficient *r* = 0.634; *p* < 0.0001). Our results clearly show sex differences in degranulation activity of intrahepatic NK cells of HBV-infected patients. This probably contributes to the ability of females to better deal with HBV disease.

## 1. Introduction

The liver is an immune-privileged organ in which antigen-rich blood is pressed through a network of microscopic vessels called sinusoids where blood is scanned by intrahepatic (IH) immune cells. IH lymphocyte population is selectively enriched in natural killer (NK) cells, which play critical roles in controlling both viral hepatitis infections and liver tumorigenesis.

Major sex differences in hepatitis B virus (HBV) infection and the male susceptibility for hepatitis-related hepatocellular carcinoma (HCC) have been described. However, distinct mechanisms have remained enigmatic. In fact, the prevalence, intensity, and severity of HBV disease itself are consistently higher in men than in women [1–3]. The higher incidence of HBV in men for sure contributes to sex differences in occurrence of HCC, but even among HBsAg-positive individuals, liver cancer mortality is two times higher in males compared to females [1]. Sex-specific differences in exposure to risk factors, such as alcohol consumption or drug use in male population, do not fully explain the greater severity of HBV disease and the higher occurrence of HCC in males compared to females. For instance, same sex differences are also observed during animal experiments. Understanding the mechanisms that enable females to better deal with HBV disease and to reduce their risk of developing HCC needs to be elucidated.

It is known that females often exhibit greater humoral and cell-mediated immune responses to infection than do males [1, 4, 5]. Similarly, numerous in vitro and in vivo experiments have demonstrated that sex hormones directly or indirectly affect and modify the actions of immune cells [6]. The female and male livers show considerable sexual dimorphism, and when taking into account that sex hormones are notably metabolised in the liver, the effects of sex hormones on IH immune cell actions are expectable.

Therefore, the objective of this study was to investigate degranulation activity of peripheral and IH-NK cells during chronic hepatitis B infection with a focus on sex differences.

## 2. Methods

### 2.1. Patients

One hundred twenty-five patients included in this study were prospectively selected prior to any treatment (Department of Gastroenterology and Hepatology, Grenoble University Hospital). HBV-infected patients (*n* = 43, 63% men) were positive for anti-HBV antibody (tested by ELISA 3, Ortho Diagnostic Systems, USA), positive for HBV DNA in serum as measured by qPCR (Amplicor HCV, Roche Diagnostic Systems), and negative for human immunodeficiency virus (HIV) and, HCV infections and did not have any autoimmune hepatitis (AIH) antibody. HCV-infected patients (*n* = 82, 52% men) were positive for anti-HCV antibody (tested by ELISA 3, Ortho Diagnostic Systems, USA), positive for HCV RNA in serum as measured by RT-qPCR (Amplicor HCV, Roche Diagnostic Systems), and negative for HIV and HBV infections and did not have AIH antibody. Alcohol consumption of patients was lower than 30 g/day in men and 20 g/day in women. The main characteristics of all patients are described in Table S1 in Supplementary Material available online at https://doi.org/10.1155/2017/3214917.

Liver biopsies were divided into two parts: one part for histological examination and the other part for immunological analyses. Histological examination was assessed by experienced liver pathologists. Paired blood and liver samples were obtained in 16 HBV-infected patients (Supplementary Figure 1C).

The study was performed in accordance with the Declaration of Helsinki and French legislation and received approval of the Grenoble University Hospital ethical committee (03/APTF/1). All study participants provided written informed consent.

### 2.2. Flow Cytometric Analysis

Immediately after the liver biopsy procedure, cells were recovered by mechanical disruption and stained for flow cytometric analysis as described previously [7]. Similarly, peripheral blood cells were immunostained. Live/dead cells were discriminated by a Zombie UV™ Fixable Viability Kit. The following antibodies were used for surface staining: CD45 (APC/Cy7, Clone HI30, BioLegend), CD3 (PerCP/Cy5.5, Clone UCHT1, BioLegend), and CD56 (APC, Clone HCD56, BioLegend). Surface staining of CD107a (Pacific Blue, LAMP-1, Clone H4A3, 0.25 *μ*g/sample) was used to study degranulation activity [8–13]. Data were acquired on BD-LSRII flow cytometer (BD Biosciences), collected with BD FACSDiva 6.3.1 software, and analyzed using FCS Express V3 and V6 software.

### 2.3. K562 Target Cell Activation

IH immune cells were incubated for 3 h at 37°C with or without K562 target cells (cell : target = 1 : 1) in the presence of monensin (Sigma). Degranulation activity was monitored by detection of cell surface CD107a.

### 2.4. NK Cell Lines

Cell lines were cultured in RPMI 1640 medium supplemented with 1% of antibiotics (Pen Strep, Life Technologies), recombinant IL2, and 1 mM sodium pyruvate and with 10% fetal bovine serum for KHYG1 and NK92 cell lines and with 10% heat-inactivated human serum, type AB for an NKL cell line. All sex hormones: estrone (E9750), *β*-estradiol (E8875), testosterone (T1500), and 4,5*α*-dihydrotestosterone (A8380), were purchased from Sigma. Hormones were added to the culture media in a concentration of 10 nM for 24 h before the stimulatory experiment by ±K562 target cells.

### 2.5. Measurements of Hormone Levels

Levels of estradiol and testosterone were measured in serum by commercially available ELISA kits (Abcam, UK), according to manufacturer's protocols.

### 2.6. Statistical Methods

Analyses were performed using the statistical software GraphPad Prism 6 (GraphPad Software, CA, USA). Gaussian distribution was tested by the D'Agostino-Pearson omnibus normality test. The *t*-test was used in the case of normal distribution of data and the nonparametric Mann-Whitney test in the case of nonnormality. The Wilcoxon matched-pairs signed-rank test was used to test differences between K562 nonstimulated and K562 stimulated cells. Pearson's correlation coefficients were used for the linear relationship between two variables.

## 3. Results

**Figure 1:**
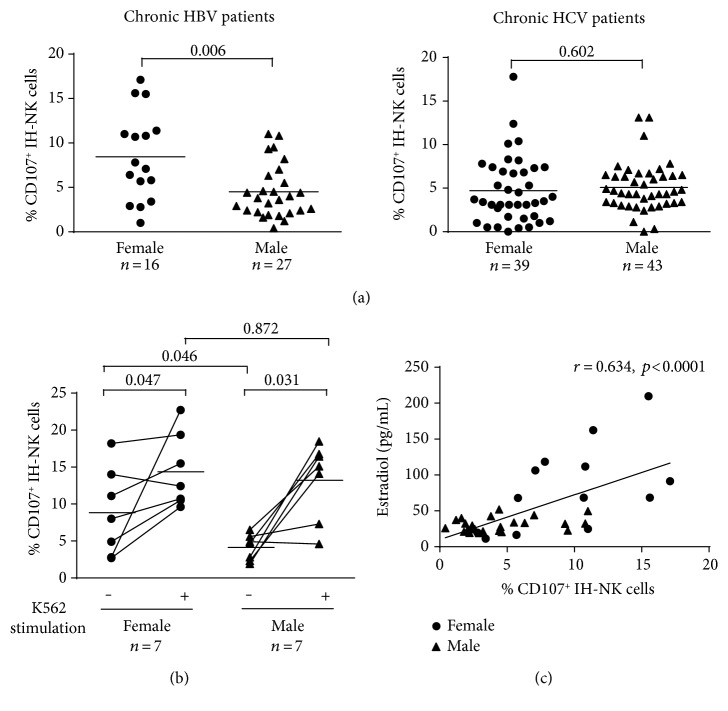
(a) Degranulation activity of IH-NK cells of chronic HBV- and HCV-infected patients directly after the recovery of liver biopsies. Gaussian distribution was tested by the D'Agostino-Pearson omnibus normality test. The Mann-Whitney test was used to test the HBV cohort and the *t*-test to test the HCV cohort. (b) Frequencies of degranulating IH-NK CD56^Dim^ cells of chronically HBV-infected patients stimulated in vitro by ±3 h of K562 target cells. The Mann-Whitney test was used to compare between females and males, while the Wilcoxon matched-pairs signed-rank test was used to compare before and after K562 stimulation. (c) Correlation of estradiol serum levels with spontaneous degranulation capacity of intrahepatic NK cells of chronically HBV-infected patients. To test significance, Pearson's correlation coefficients were used. Each symbol represented a patient and mean values are indicated by lines.

Immune cells from blood and from fresh liver biopsies of infected patients (Supplementary Table 1) were analyzed by flow cytometry. Among CD45^high+^ population, NK cells were identified (Supplementary Figure 1A). To investigate the NK cell cytolytic properties, we determined the CD107a expression, which is considered a marker of degranulation, that is, release of lytic granules toward the target cells [12, 13]. Mean percentage of CD107a^+^ IH-NK cells was significantly higher in liver biopsies from HBV-infected females (*n* = 16) compared to HBV-infected males (*n* = 27), with a frequency of CD107a^+^ IH-NK 8.4 ± 1.2% in females compared to 4.5 ± 0.6% in males (Figure 1(a)). In HCV-infected patients, the frequency of CD107^+^ IH-NK cells was similar between females (4.7 ± 0.6%, *n* = 39) and males (5.1 ± 0.4%, *n* = 43).

When the cohorts were compared regardless of gender, we observed no difference in the frequency of CD107^+^ intrahepatic NK cells in HBV patients compared to HCV patients. However, the frequency of CD107^+^ intrahepatic NK cells was significantly higher in HBV-infected women than in HCV-infected women (*p* = 0.0096) or men (*p* = 0.0206) and corresponded to degranulation activity that can be observed in patients with AIH (Supplementary Table 2).

To exclude the possibility that observed sex differences in the functional degranulation activity of NK cells were caused by differences in severity of HBV disease, 16 HBV-infected males matched for age and severity of liver injury (ALT levels, METAVIR activity grade, and fibrosis stage) were compared with 16 females (Supplementary Table 3). Similarly as in the whole cohort, the percentage of CD107a^+^IH-NK cells was 2.2-fold higher in females than in males (*p* = 0.0031) (Supplementary Figure 1B). On the other hand, the frequency of CD107^+^ NK cells in blood samples was very low, both among females (0.64 ± 0.16%) and among males (0.63 ± 0.14%) (Supplementary Figure 1C). A similar low frequency of spontaneously degranulating NK cells was found previously in blood of HCV-infected patients, showing that the degranulation process of NK cells occurs mainly in the liver [13]. Interestingly, our preliminary results showed that even the percentage of IFN*γ*^+^ IH-NK cells is 2.6-fold higher in HBV-infected females than in males (*p* = 0.0044) (Supplementary Figure 1D), while no sex difference in IFN*γ*^+^ IH-NK cells is observed in the cohort HCV-infected patients. All together, these results indicate that sex differences in degranulation activity of NK cells are specific for the HBV-infected liver.

To analyze whether the stimulated degranulation capacity of IH-NK cells differs between sexes, liver biopsies of HBV-infected females (*n* = 7) and males (*n* = 7) were divided in two parts and immune cells of one part were incubated for 3 hours with K562 target cells. In accordance with the results described above, the mean frequency of CD107^+^ IH-NK cells without any stimulation was significantly higher in females compared to males (*p* = 0.033). This difference was statistically significant specifically in CD107^+^ CD56^Dim^ IH-NK cell population (*p* = 0.046) (Figure 1(b), Supplementary Table 4). Interestingly, upon stimulation, we observed a 3.2-fold increase in the mean frequency of CD107^+^ CD56^Dim^ IH-NK cells of males (*p* = 0.031) but only a 1.6-fold increase in that of females (*p* = 0.047) (Figure 1(b)). Thus, after in vitro K562 stimulation, the frequencies of CD107^+^ CD56^Dim^ IH-NK cells did not differ between the sexes (*p* = 0.872) underlining that the degranulation capacity (in vitro-stimulated degranulation) of IH-NK cells is equal in males and females. In summary, these results suggest that NK cells in the liver of HBV-infected women are specifically activated.

To investigate if the activity of NK cells is modified by sex hormones, we analyzed serum from chronically HBV-infected patients by ELISA. A strong correlation was observed between the spontaneous degranulation activity of IH-NK cells and levels of estradiol (Figure 1(c), *p* < 0.0001) while no correlation was observed in testosterone levels (Supplementary Figure 1E). Interestingly, no other correlations were observed between the spontaneous degranulation activity of IH-NK cells and age, ALT levels, METAVIR activity grade, fibrosis stage, or viral load in HBV-infected patients (Supplementary Table 1).

To clarify if NK cell functions are dependent on sex hormones, we used three different well-established human NK cell lines: KHYG1 (originally from female), NK92, and NKL (originally from male), and stimulated them by sex hormones. Even though NK cells express sex hormone receptors [14–16], no direct effect of estradiol or other sex hormones on degranulation activity of human NK cell lines was observed (Supplementary Figure 1F). However, the fact that NK cell lines are of peripheral blood origin and HBV infection was missing in this scenario makes drawing conclusions about the possible indirect action of estrogens on NK cells difficult.

Taken together, our results show that during HBV infection, the degranulation activity of IH-NK cells is associated with estradiol levels.

## 4. Discussion

Our results clearly show sex differences in the spontaneous degranulation activity of IH-NK cells of HBV-infected patients in correlation with levels of circulating estradiol.

Previously, in vitro assessment of degranulation activity after K562 stimulation of NK cells from the blood of healthy subjects showed higher stimulated activity in men compared to women [17], depending on menstrual cycle [18], but spontaneous activity of NK cells was not determined. In our study, we did not observe sex differences in the spontaneous activity of circulating NK cells of HBV-infected patients, but in vitro stimulation of IH-NK cells by K562 target cells showed a 3.2-fold increase in degranulation in men compared to only a 1.6-fold increase in women. In fact, as IH-NK cells in the female liver were already activated, further in vitro activation was not as effective as in males. Therefore, it is important to distinguish spontaneous and in vitro-stimulated degranulation activity of NK cells when interpreting the results.

Higher immune responses in females not only can result in faster clearance of infections but also contribute to increased susceptibility to autoimmune diseases [1, 4, 5]. The direct role of overactivated NK cells in the liver damages occurring during the course of autoimmune hepatitis has been described [19]. However, in our cohort of HBV-infected women, we did not observe higher liver damages even though the frequency of activated IH-NK cells was significantly increased compared to that of men. This is probably due to the fact that the degranulation activity of IH-NK cells is not increased constantly in HBV-infected females but correlates with estradiol levels, which rise and fall during the menstrual cycle with a peak of estradiol level during only 2-3 days during the late follicular phase. The association with levels of circulating estradiol also explains high heterogeneity in frequency of CD107^+^ IH-NK cells in HBV-infected females (Figure 1(a)).

The protective effects of estrogen are thought to enable women to clear the HCV infection and thus progress slower to the disease than in men [20] and NK cells contribute to this difference, since NK p46 expression on NK cells is higher in HCV-infected females compared to males [21]. In HBV infection, higher spontaneous degranulation activity of NK cells in females was never reported probably due to the fact that research is mainly focused on circulating NK cells which differ radically from NK cells in the liver where these immune cells have unique phenotypic features and functional properties [19]. Moreover, during chronic HBV infection, a specific cross talk is established between different immune cells in the infected liver. It has been shown that for instance, impaired interactions between plasmacytoid dendritic cells (pDCs) and NK cells reduce immune control of HBV and lead to chronic infection [22]. Interestingly, pDCs are known to be strongly positively regulated by estrogens [23]. Therefore, one plausible explanation is that estradiol-activated pDC may increase degranulation activity of NK cells in HBV-infected women. However, the exact mechanism on how estrogens stimulate degranulation activity of NK cells in the liver needs to be further investigated.

## 5. Conclusions

In this study, we provide evidence that the frequency of CD107^+^ IH-NK cells in the female liver is higher compared to that in males during chronic HBV infection and correlates with the estradiol levels. This phenomenon can contribute to sex-related differences in intensity and severity of HBV disease.

## Supplementary Material

Supplementary Information: Supplemetary Figure 1. Gating strategy and degranulation of NK cells. A) Gating strategy to investigate degranulation activity of NK cells. Dead cells were excluded and immune cells were identified according their FSC and SSC parameters (left panel). Cells were further gated based on their CD45^high+^ expression and NK cells (CD56^+^CD3^−^), NKT cells (CD56^+^CD3^+^) and T cells (CD56^−^CD3^+^) were selected. To study degranulation activity, surface expression of CD107 was analysed. B) Degranulation of NK cells in liver of selected chronic HBV-infected patient cohort. C) Degranulation of NK cells from blood of chronic HBV-infected patients. D) Frequency of IFN gamma^+^ NK cells in the liver of chronic HBV-infected patients. In this cohort, the liver cell suspension was stained by surface antibodies: CD45, CD3, CD56, then fixed, permeabilized, and stained for IFN γ (BV421, Clone 4S.B3) E) Correlation of testosterone serum levels with spontaneous degranulation capacity of intrahepatic NK cells of chronic HBV-infected patients. F) Frequencies of CD107+ human NK cell lines cultured in media −/+ sex hormones for 24h and stimulated by −/+ 3h of K562 target cells. Human NK cell lines: KHYG1 (originally from female (F)), NK92 and NKL (originally from male (M)). Cell line experiments were performed in duplicates and repeated at least three times. Gaussian distribution was tested by D'Agostino & Pearson omnibus normality test and non-parametric Mann-Whitney test was used to test HBV cohort; Supplementary Table 1. Demographic and clinical parameters of cohorts. Demographic and clinical parameters of chronically HBV and HCV-infected patients. ^*^Data are Mean ± SE, ^#^ Data are expressed as Median; Supplementary Table 2. Degranulation of intrahepatic natural killer cells during liver diseases. Spontaneous degranulation activity of intrahepatic natural killer cells during chronic hepatitis B (HBV), chronic hepatitis C (HCV), Nonalcoholic steatohepatitis (NASH) and Autoimmune hepatitis (AIH). Data are Mean ± SE; Supplementary Table 3. Demographic and clinical parameters of HBV cohort. Demographic and clinical parameters of chronically HBV-infected patients (n=16). Data are Mean ± SE; Supplementary Table 4. Degranulation of intrahepatic CD56 Bright versus Dim natural killer cells. Frequency of intrahepatic CD107a^+^ NK cells of chronically HBV-infected females (F, n=7) and males (M, n=7). Without (Non-stimulated) or with (Stimulation) K562 target cells. Data are Median [Min-Max].

## Abbreviations

AIH: Autoimmune hepatitis
HBV: Hepatitis B virus
HCC: Hepatocellular carcinoma
HCV: Hepatitis C virus
IH: Intrahepatic
NK: Natural killer.
