# Supplementary material for: Sex Differences in Spontaneous Degranulation Activity of Intrahepatic Natural Killer Cells during Chronic Hepatitis B: Association with Estradiol Levels
Source: Mediators Inflamm. 2017 Apr 2;2017:3214917. doi: 10.1155/2017/3214917 (PMC5392396; doi:10.1155/2017/3214917)
Supplement: Supplementary file 1 — Supplementary Information: Supplemetary Figure 1. Gating strategy and degranulation of NK cells. A) Gating strategy to investigate degranulation activity of NK cells. Dead cells were excluded and immune cells were identified according their FSC and SSC parameters (left panel). Cells were further gated based on their CD45high+ expression and NK cells (CD56+CD3−), NKT cells (CD56+CD3+) and T cells (CD56−CD3+) were selected. To study degranulation activity, surface expression of CD107 was analysed. B) Degranulation of NK cells in liver of selected chronic HBV-infected patient cohort. C) Degranulation of NK cells from blood of chronic HBV-infected patients. D) Frequency of IFN gamma+ NK cells in the liver of chronic HBV-infected patients. In this cohort, the liver cell suspension was stained by surface antibodies: CD45, CD3, CD56, then fixed, permeabilized, and stained for IFN γ (BV421, Clone 4S.B3) E) Correlation of testosterone serum levels with spontaneous degranulation capacity of intrahepatic NK cells of chronic HBV-infected patients. F) Frequencies of CD107+ human NK cell lines cultured in media −/+ sex hormones for 24h and stimulated by −/+ 3h of K562 target cells. Human NK cell lines: KHYG1 (originally from female (F)), NK92 and NKL (originally from male (M)). Cell line experiments were performed in duplicates and repeated at least three times. Gaussian distribution was tested by D'Agostino & Pearson omnibus normality test and non-parametric Mann-Whitney test was used to test HBV cohort; Supplementary Table 1. Demographic and clinical parameters of cohorts. Demographic and clinical parameters of chronically HBV and HCV-infected patients. *Data are Mean ± SE, # Data are expressed as Median; Supplementary Table 2. Degranulation of intrahepatic natural killer cells during liver diseases. Spontaneous degranulation activity of intrahepatic natural killer cells during chronic hepatitis B (HBV), chronic hepatitis C (HCV), Nonalcoholic steatohepatitis (NASH) and Autoimm [file 3214917.f1.pdf]

Supplementary Information

Sex differences in spontaneous degranulation activity of intrahepatic natural killer cells during chronic hepatitis B; association with estradiol levels

Zuzana Macek Jilkova[1, 2], Thomas Decaens[1, 2, 3], Alice Marlu [3], Hélène Marche[1, 2], Evelyne Jouvin-Marche [1,2] and Patrice N Marche[1, 2]

[1] Université Grenoble-Alpes, IAB, F38000 Grenoble, France; [2] INSERM U1209, F-38000 Grenoble, France; [3] CHU-Grenoble Alpes, Département d'Hépato-Gastro-Entérologie, F30700 La Tronche.

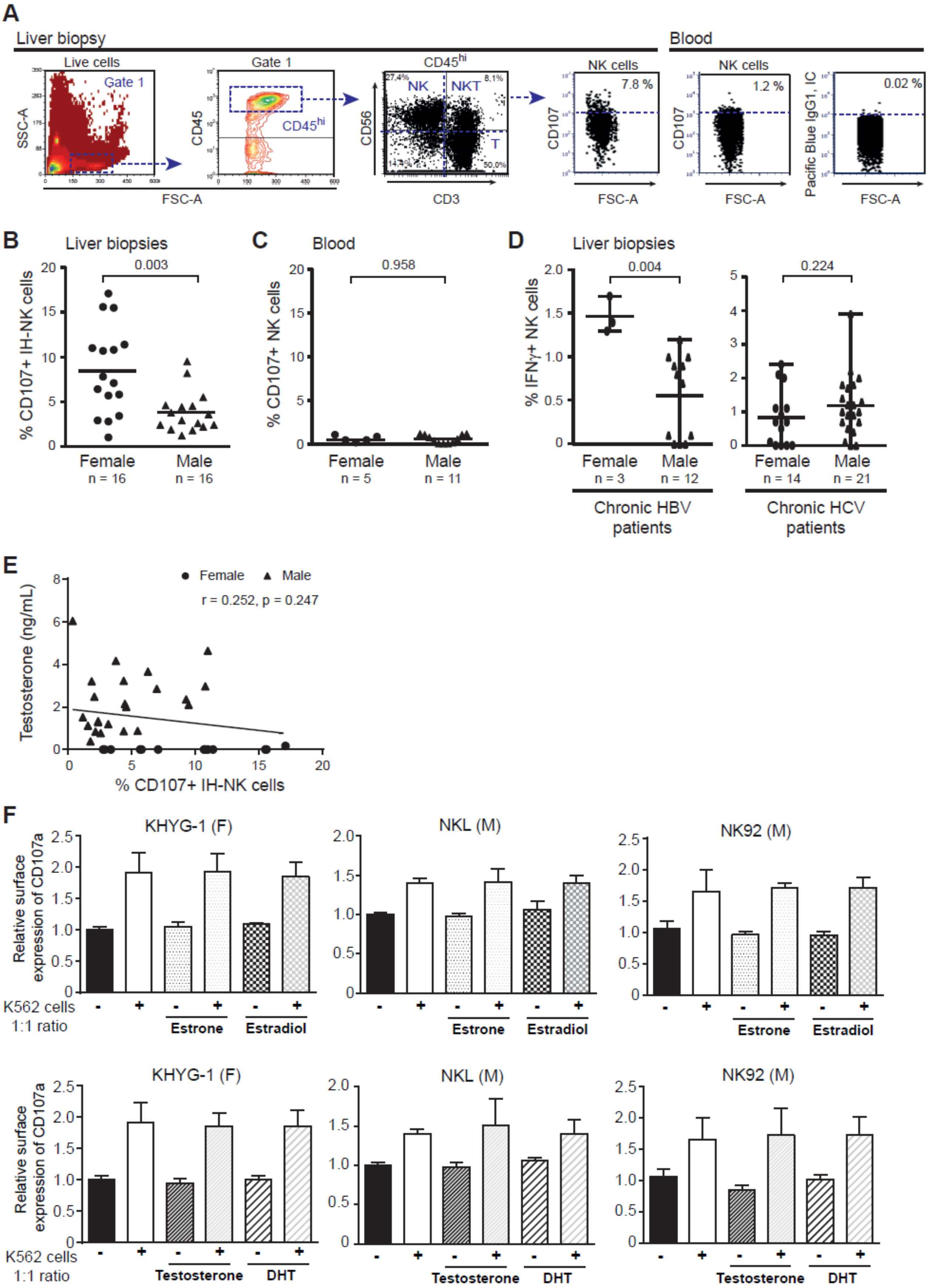

A
Liver biopsy
Blood
Live cells
Gate 1
SSC-A
FSC-A
CD45
CD45hi
NK
NKT
T
CD56
CD3
NK cells
7.8 %
CD107
1.2 %
0.02 %
Pacific Blue IgG1, IC
B
Liver biopsies
0.003
% CD107+ IH-NK cells
Female
n = 16
Male
n = 16
C
Blood
0.958
% CD107+ NK cells
Female
n = 5
Male
n = 11
D
Liver biopsies
0.004
0.224
% IFNγ+ NK cells
Female
n = 3
Male
n = 12
Chronic HBV patients
Female
n = 14
Male
n = 21
Chronic HCV patients
E
Female
Male
r = 0.252, p = 0.247
Testosterone (ng/mL)
% CD107+ IH-NK cells
F
KHYG-1 (F)
NKL (M)
NK92 (M)
Relative surface expression of CD107a
K562 cells 1:1 ratio
Estrone
Estradiol
Testosterone
DHT

Supplemetary Figure 1:

Gating strategy and degranulation of NK cells

A) Gating strategy to investigate degranulation activity of NK cells. Dead cells were excluded and immune cells were identified according their FSC and SSC parameters (left panel). Cells were further gated based on their $CD45^{high+}$ expression and NK cells ($CD56^{+}CD3^{-}$), NKT cells ($CD56^{+}CD3^{+}$) and T cells ($CD56^{-}CD3^{+}$) were selected. To study degranulation activity, surface expression of CD107 was analysed. **B)** Degranulation of NK cells in liver of selected chronic HBV-infected patient cohort. **C)** Degranulation of NK cells from blood of chronic HBV-infected patients. **D)** Frequency of IFN $gamma^{+}$ NK cells in the liver of chronic HBV-infected patients. In this cohort, the liver cell suspension was stained by surface antibodies: CD45, CD3, CD56, then fixed, permeabilized, and stained for IFN γ (BV421, Clone 4S.B3) **E)** Correlation of testosterone serum levels with spontaneous degranulation capacity of intrahepatic NK cells of chronic HBV-infected patients. **F)** Frequencies of CD107+ human NK cell lines cultured in media -/+ sex hormones for 24h and stimulated by -/+ 3h of K562 target cells. Human NK cell lines: KHYG1 (originally from female (F)), NK92 and NKL (originally from male (M)). Cell line experiments were performed in duplicates and repeated at least three times. Gaussian distribution was tested by D'Agostino & Pearson omnibus normality test and non-parametric Mann-Whitney test was used to test HBV cohort.

	Chronic HBV infection			Chronic HCV infection		
	**Females**	**Males**	*p-value*	**Females**	**Males**	*p-value*
N	16	27		39	43	
Age (yr)*	44.5 ± 2.9	40 ±2.6	*0.279*	53.8 ±1.8	50.0 ±1.4	*0.116*
ALT (IU/L)*	39.0 ± 4.2	58.4 ±10.9	*0.217*	69.7 ±10.5	107 ±13.8	*0.033*
AST (IU/L)*	36.8 ± 4.1	39.9 ±6.0	*0.921*	48 ±6.2	68 ±11.1	*0.117*
Viral load (IU/mL)#	7574	7435	*0.188*	646000	869850	*0.185*
Metavir activity						
A0	4	6		0	0	
A1	9	18		21	14	
A2	3	2		14	21	
A3	0	1		4	8	
Metavir fibrosis						
F0	1	5		0	0	
F0/1	3	1		0	0	
F1	11	15		22	19	
F2	1	2		13	15	
F3	0	3		3	5	
F4	0	1		1	4	

Supplementary Table 1

Demographic and clinical parameters of cohorts.

Demographic and clinical parameters of chronically HBV and HCV-infected patients. *Data are Mean ± SE, # Data are expressed as Median.

	Frequency of $CD107^+$ intrahepatic NK cells		
Etiology (n=F/M)	Females	Males	*p-value*
Chronic HBV (16/27)	8.4 ± 1.2	4.5 ± 0.6	*0.0061*
Chronic HCV (39/43)	4.7 ± 0.6	5.1 ± 0.4	*n.s.*
NASH (8/11)	4.6 ± 0.5	3.5 ± 0.3	*n.s*
AIH (15/2)	8.2 ± 0.9	9.2± 0.8	*n.s*

Supplementary Table 2

Degranulation of intrahepatic natural killer cells during liver diseases.

Spontaneous degranulation activity of intrahepatic natural killer cells during chronic hepatitis B (HBV), chronic hepatitis C (HCV), Nonalcoholic steatohepatitis (NASH) and Autoimmune hepatitis (AIH). Data are Mean ± SE.

	**Females** n = 16	**Males** n = 16	*p-value*
Age (yr)	44.5 ± 2.9	43.7 ± 2.4	*0.82*
ALT (IU/L)	39.0 ± 4.2	40.2 ± 3.9	*0.83*
AST (IU/L)	36.8 ± 4.1	30.8 ± 3.4	*0.13*
Metavir activity			
A0	4	5	
A1	9	10	
A2	3	1	
Metavir fibrosis			
F0	1	3	
F0/1	3	1	
F1	11	10	
F2	1	2	

Supplementary Table 3

Demographic and clinical parameters of HBV cohort.

Demographic and clinical parameters of chronically HBV-infected patients (n=16). Data are Mean ± SE.

	Non-stimulated			Stimulated		
	**F**	**M**	*p-value*	**F**	**M**	*p-value*
CD 107^{+} NK cells	7.8 [2.8-17.1]	4.5 [1.8-6.9]	*0.033*	12.6 [9.7-20.5]	14.8 [4.9-30.74]	0.965
CD107^{+} CD56^{Bright} NK cells	6.7 [0-38.9]	3.5 [0-11.1]	*0.104*	9.1 [0-44.4]	8.8 [7.7-40.0]	0.592
CD107^{+} CD56^{Dim} NK cells	8.0 [2.7-18.2]	4.7 [1.9-6.5]	*0.046*	12.2 [9.6-22.7]	13.3 [15.1-4.6]	0.872

Supplementary Table 4

Degranulation of intrahepatic CD56 Bright versus Dim natural killer cells.

Frequency of intrahepatic CD107a^{+} NK cells of chronically HBV-infected females (F, n=7) and males (M, n=7). Without (Non-stimulated) or with (Stimulation) K562 target cells. Data are Median [Min-Max].
